# The impact of access to electricity on mental health in conflict-affected territories: An exploratory study in Gaza

**DOI:** 10.1177/00207640231194479

**Published:** 2023-09-07

**Authors:** Mazen AbuQamar, Dalia Eltayyan, Irina Kuznetsova, Surindar Dhesi, Jonathan Catling, Raya AL-Dadah, Mahmoud Saad, Mohammad Abuhaiba

**Affiliations:** 1 Faculty of Applied Medical Sciences (FAMS), Al Azhar University-Gaza, Palestinian Territory, occupied; 2 Islamic University of Gaza (IUG), Palestinian Territory, occupied; 3 School of Geography, Earth and Environmental Sciences, University of Birmingham, UK; 4 School of Psychology, University of Birmingham, UK; 5 Department of Mechanical Engineering, University of Birmingham, UK; 6 Mechanical Engineering Department, Islamic University of Gaza (IUG), Palestinian Territory, occupied

**Keywords:** Mental health, Gaza, electricity, depression, conflict

## Abstract

**Background:**

Access to affordable and clean energy is an essential component of the Sustainable Development Goals and a determinant of physical and mental health. However, the occupied Palestinian territory, the Gaza Strip, has experienced prolonged issues with electricity, water and gas supplies. This has significantly impacted on daily life and the area is on the verge of disaster. This research focused on the mental health effects of the lack of electricity in Gaza which have not been previously documented.

**Methods:**

A cross-sectional analytic approach was adopted. A survey was administrated face-to-face with respondents from 350 participating households. Inferential statistical analysis was used to examine the relationship between the domains of anxiety, depression, wellbeing and electricity supply factors. A multiple linear regression model was also utilised.

**Results:**

There is a highly statistically significant link between continuity of electricity and level of anxiety (*p* < .001). The same effect was reported in the level of depression, and a higher level was observed among residents with an intermittent or disrupted electricity supply, with a statistically significant link between the level of depression and continuity of electricity.

**Conclusion:**

Electricity issues, especially when combined with other stressors associated with living in Gaza, lead to serious mental health concerns. Urgent attention must be given to developing sustainable, reliable and affordable energy supplies for short- and long-term health and community development.

## Introduction

Access to electricity is one of the essential components of Sustainable Development Goal (SDG) 7, ‘Affordable and clean energy’. However, according to the International Energy Agency, the world is not on track to achieving SDG 7 as 759 million people still lack electricity. Half of them live in fragile and conflict-affected settings (International Renewable Energy Agency, 2021). Home to nearly two million people, including 1.4 million refugees, the blockaded Gaza Strip has long struggled with severe electricity shortages, with only 38% of electricity needs met. On average, power was off for occupants of the Gaza Strip for 11 hours per day in 2021 (OCHA, n.d.). In 2017 and 2018, due to disputes between the de facto authorities in Gaza and the West Bank-based Palestinian Authority, electricity was available on average only for 7 hours per day (OCHA, n.d.). The inadequate electricity supply has persisted in the Gaza Strip for decades (Ismail et al., 2013).

According to the UN Office for Coordination of Humanitarian Affairs, this acute energy crisis pushes the area to the verge of disaster with severe implications for the health, water and sanitation sectors. This electricity crisis coupled with the continuous conflict causes high levels of stress that affects people’s physical, and mental health and wellbeing. The lack of electricity significantly affects the mental health of refugees and permanent residents in Gaza as they live in challenging environmental conditions and often do not have access to alternative sources of electricity. Education is hindered by the electricity shortage as well, especially in winter. For example, 83.5% of students in Gaza reported that their studies were compromised due to the cut-off of electricity and shortage of gas (Thabet & Thabet, 2015).

Until recently, there were no studies focused on understanding the impact of the lack of electricity on mental health in conflict-affected areas.

The impact of housing on health is widely recognised (Braubach, 2011, Singh et al., 2019). The World Health Organization (WHO) suggests focusing on priorities related to several issues, including housing quality, access to inequality, building regulations, etc (Braubach, 2011). However, access to safe and accessible electricity was not explicitly stated. A recent study highlights the effect of a deteriorated socioeconomic situation on mental health (Hamad et al., 2020), however there are currently no studies which focus specifically on understanding the impact of lack of electricity on mental health in conflict-affected areas.

A mixed-method systematic review of the impact of housing on the health of people with refugee backgrounds revealed links between housing and mental health relating to housing conditions, insecurity and mobility, discrimination in accessing housing, overcrowding, and a sense of safety and social connections (Ziersch & Due, 2018). A systematic review on the impact of access to electricity on health in low- and middle-income countries found that ‘gaining access to electricity was linked to reduced mortality and morbidity, particularly from respiratory conditions, as well as increased care-seeking and quality of care received overall, resulting from changes to the individual, household, community, and institutional determinants of health’ (Irwin et al., 2020). However, only one paper from the review’s sample referred to mental health (Ibrahim et al., 2016). The study identified a correlation between power outages and generalised anxiety disorders in a cross-sectional survey of university students in Ghana.

Over half of the population in Gaza suffer from the lack of electricity supply, clean water and cooking gas which negatively impacts their wellbeing (Abu-Rmeileh et al., 2012). McNeely et al.’s (2014) research in occupied Palestinian territory demonstrated correlations between human insecurity, resource inadequacy (including the inadequacy of housing), and feelings of depression, trauma-related stress and wellbeing. The mental health of the population in the Gaza Strip is massively affected by the siege and blockade (Madianos et al., 2011), especially among ‘older age fathers who live in a refugee camp and unemployed and living in poor families’ (Thabet & Thabet, 2015). At the same time, as Makkawi (2015) and Hammad and Tribe (2020) noticed, there is a lack of continued studies on mental health in the Gaza Strip as public health research on Palestinians has been primarily conducted in the West Bank.

This research aims to fill this lacuna by exploring how the Gaza population’s mental health is affected by the lack of electricity in the Gaza Strip.

## Methods

### Participants

350 households participated in the study, these only included people over 18 years of age. The survey was administered in a semi-stratified fashion to allow for a range of locations and following the demographic patterns of the population in terms of gender and age based on the 2021 the World bank Open data (n.d.). Opportunity sampling was utilised in selecting the participants from the households. To mitigate any disparities in sampling, the survey was administrated in different time of the day to include both employed and unemployed populations. The survey was administrated during face-to-face structured interviews. Interviews with men were mostly conducted by a male data collector, and interviews with women participants by a female data collector. Approximately 40% of participants lived in the Gaza (G) governorate, 21% in Khan-Younis (KH), 20% in the North governate (NG), 13% in Rafah (RG), and 7% in the Middle governorate (MG). The demographics of participants are presented in the Supplemental materials. The sample in general represented the population well in terms of gender with 49% of male participants (49.87% according to the World Bank).

According to Palestinian Central Bureau of Statistics, Gaza Population in 2022 is 2,300,000. 52% of them are adults over 18 years, Population over 18 yes is 1,212,100. According to the software sample calculator the sample size is 385. We collected data from 350 participants from all Gaza governorates (5 governorates based on proportional population size in each governorate). The sample considers the proportion of city, camp and village in each governorate.

### Measures

A survey that assessed for demographics, living environment and physical health was administered. In addition, three standardised questionnaires were utilised to assess Mental health. Patient Health Questionnaire (PHQ-9; Kroenke et al., 2009) was used as a self-report measure of depression severity. The questionnaire focuses on diagnostic criteria for depression (DSM-IV), assessing severity via nine questions on a scale from experiencing a problem ‘not at all’ (0) to ‘nearly every day’ (3) over the last 2 weeks. Higher scores represent higher depression severity, the highest possible score is 27. Internal consistency was reviewed with a Cronbach’s α coefficient of 0.86, with good test-retest reliability. Beard et al. (2016) identified good convergent and discriminant validity in a psychiatric sample. Manea et al. (2012) identified an optimal cut-off score of 10 when diagnosing (moderate to severe) depression with the PHQ-9 with 88% sensitivity and 88% specificity.

Generalised Anxiety Disorder-7 (GAD-7; Spitzer et al., 2006) was used as a self-report measure of anxiety. Seven symptoms of anxiety based on diagnostic criteria (DSM-IV) are measured, from the problem bothering an individual ‘not at all’ (0) to ‘nearly every day’ (3) over the last 2 weeks. Higher scores represent higher anxiety severity, the highest possible score is 21. Cronbach’s α coefficient for internal consistency was measured at 0.92, and test–retest reliability correlated at 0.83, and good criterion and procedural validity was shown. Spitzer et al. (2006) identified a cut-off score of 10 when diagnosing (moderate to severe) anxiety with the GAD-7 with 89% sensitivity and 82% specificity.

5-item World Health Organization Well-Being Index (WHO-5) is among the most widely used questionnaires assessing subjective psychological well-being. Since its first publication in 1998, the WHO-5 has been translated into more than 30 languages and has been used in research studies all over the world. The WHO-5 is a short questionnaire consisting of 5 simple and non-invasive questions, which tap into the subjective well-being of the respondents. The WHO-5 has high validity, is a sensitive and specific screening tool for depression, and it is suitable for use across study fields is very high. The scale has adequate validity as a screening tool for depression and has been applied successfully across a wide range of study fields (Topp et al., 2015).

### Ethics approval

This study was approved by the Ethical Committee of the University of Birmingham (ERN_20-0535) on the 2^nd^ of November 2020 and by the Helsinki committee in Gaza. Participation was voluntary and anonymous. The participants provided informed consent and had a right to withdraw from the study.

## Results

Only 16.3% of respondents had a continuous electricity supply, and 81.4% of them had an intermittent supply, while 2.3% did not have any electricity supply. The households of 94.3% of the respondents were connected to a mains electricity supply, and 39.7% were using street generators that they use during power cuts. For lighting, only 64.3% (225 HH) were using battery power, and only 8.3% (29 HH), 10.3% (36 HH) of them were using kerosene lamps and naked flame (candles) for lightening respectively.

The abilities of the population to access some generators and some other separate sources of energy are low as about 70% of respondents live in absolute poverty (<1974 New Israeli Shekel – NIS), 10% in relative poverty (<2493 NIS), and 22% in non-poverty.

### Clinical outcomes

Anxiety (GAD-7) and depression (PHQ-9) were categorised according to their severity; the following cut-offs were utilised: Score 0–4: Minimal Anxiety, Score 5–9: Mild Anxiety, Score 10–14: Moderate to severe Anxiety, Score greater than 15: Severe Anxiety. For Depression Severity: 0–4 none-minimal, 5–9 mild, 10–14 moderate, 15–19 moderately severe and 20–27 severe.

In respect to levels of anxiety, 6.9% of the participants scored at mild anxiety, while 31.1% are moderate sufferers and 62% were rated as severe sufferers of anxiety. In respect to depression, it was found that 6.9% of participants had a mild score on depression, 31.4% of them are rated as moderate sufferers, 44% of participants had a moderate to severe score and 17.7% of participants were rated as suffering from severe depression (Table 1).

**Table 1.** Anxiety and depression scores.

Anxiety score ‘severity’	*N*	%
Anxiety		
Minimal	0	0.0
Mild	24	6.9
Moderate	109	31.1
Severe	217	62.0
Depression		
None-minimal	0	0.0
Mild	24	6.9
Moderate	110	31.4
Moderate to severe	154	44.0
Severe	62	17.7

In summary, for anxiety 93% of individuals were rated moderate-severe or severe anxiety, this compares with 6% within the general population (for average data, see Löwe et al., 2008), a one sample T-test showed that the sample mean was significantly different from the population mean (*t*(349) = 42.58, *p* < .001). For depression, 44% of individuals were rated as moderate to severe or severe for depression, this compares with 5.6% for the general population (for average data on depression, see Kocalevent et al., 2013), a one sample T-test showed that the sample mean was significantly different from the population mean (*t*(349) = 46.71, *p* < .001).

There is a need for immediate initiation of pharmacotherapy and, if a severe impairment is revealed or there is poor response to therapy, expedited referral to a mental health specialist for psychotherapy and/or collaborative management.

### Effect of gender on mental health

According to gender, 48.6% of the participants were male while 51.4% were female. For the statistical analysis of the data, a MANOVA was undertaken with Mental health measures as dependent variables and gender as the independent variable. Overall, the MANOVA was non-significant (*F* < 1). Gender did not have a significant impact on any of the physical and mental health outcome measures.

### Effect of location on mental health

A second MANOVA was undertaken with mental health measures as dependent variables and location as the independent variable. Overall the MANOVA was significant (*F*(12,1035) = 4.347, *p* < .001. Individually there was a significant difference in levels of anxiety *F*(4,345) = 6.236, *p* < .001, depression *F*(4,345) = 4.225, *p* < .005, and WHO-5 *F*(4,345) = 3.726, *p* < .01.

Post-Hoc Tukey tests showed that for anxiety there were significant differences for anxiety between NG and G regions (*p* < .005), between NG and RG regions (*p* < .005), MG and RG (*p* < .05), and KH and RG (*p* < .05). Those participants within the Northern and Middle Governates were highest and those in Rafah were lowest.

For depression there were significant differences between the NG and G regions (*p* < .05) and between the MG and G regions (*p* < .05). Again, Northern and Middle Governates showed the highest levels of depression, and Gaza the lowest.

For the WHO-5 measure there was only significant differences between NG and G regions (*p* < .05) (see Table 2), with the North showing the lowest wellbeing scores and Rafah showing the highest wellbeing scores.

**Table 2.** Prevalence of anxiety, depression and wellbeing in different areas of Gaza.

Governate	Northern	Gaza	Rafah	Middle	Khan-Younis
GAD-7	10.1	7.9	7	10	9.1
PHQ-9	12.7	10.4	10.7	13.7	11.7
WHO-5	16.7	19.1	19.2	16.5	17.8

### Effect of source of electricity on mental health

Most participants were connected to the mains supplies 94.3% compared to 5.7% who were not connected to a mains supply. A MANOVA was undertaken with combined mental health indicators (anxiety, depression and wellbeing) as dependent variables and ‘connected to main electricity’ (yes/no) as the independent variable, and was non-significant (*p* > .05).

However, the MANOVA was significant (*F*(6,692) = 2.734, *p* < .001 regarding to depression. There was a significant difference in levels of depression *F*(2,349) = 3.985, *p* < 0.05, and WHO-5 *F*(2,349) = 4.063, *p* < .05. There were no observed significant differences in levels of anxiety (*p* > 0.05).

For levels of depression, post-hoc Tukey tests showed that there were significant differences between the ‘reliable source’ and ‘no electricity supply’ (*p* < .05) and between ‘intermittent supply’ and ‘no electricity supply’ (*p* < .05).

For the WHO-5 measure, post-hoc Tukey tests showed that there were significant differences between the ‘reliable source’ and ‘no electricity supply’ (*p* < .05).

The results showed a higher mean level of anxiety among those participants who experienced a constant lack of electricity and increased the level of anxiety with a statistical difference between continuity of electricity and level of anxiety. The same effect was reported in the level of depression, and a higher level was observed among those participants who experienced intermittent or disrupted electricity supply with a statistically significant level between the level of depression and continuity of electricity. Regarding wellbeing, the results showed higher wellbeing was proportional to the continuity of electricity, and there was a statistically significant difference between variables (see Table 3).

**Table 3.** Correlations between the reliability and type of power supply and anxiety, depression and wellbeing.

Domain	No.	Mean	*SD*	*t/F*	*p*-value
Shelter connected to mains electricity supply					
Anxiety					
No	20	2.73	.424	12.816	.000
Yes	330	2.25	.595		
Depression					
No	20	2.38	.650	6.381	.012
Yes	330	2.05	.562		
Wellbeing					
No	20	2.60	1.18	5.62	.020
Yes	330	3.34	1.37		
Reliability of power					
Anxiety					
Continuous supply	57	2.37	.642	3.653	.027
Intermittent	285	2.24	.583		
No power supply	8	2.74	.559		
Depression					
Electricity supply reliable	57	2.12	.585	4.626	.010
Be intermittent	285	2.04	.558		
No electricity supply	8	2.63	.699		
Wellbeing					
Electricity supply reliable	57	3.49	1.40	2.917	.055
Be intermittent	285	3.29	1.36		
No electricity supply	8	2.25	1.28		
Anxiety domain and source of power supply					
Using generators					
No	211	2.31	0.600	1.514	.133
Yes	139	2.22	0.588		
Using battery power for lighting					
No	125	2.45	0.577	1.946	0.052
Yes	225	2.23	0.605		
Using kerosene lamp					
No	321	2.27	0.596	−0.686	.493
Yes	29	2.35	0.603		
Using candles					
No	314	2.29	0.605	1.702	.090
Yes	36	2.13	0.508		
Depression domain and source of power supply					
Using generators					
No	211	2.10	0.585	1.149	.250
Yes	139	2.02	0.551		
Using battery power for lighting					
No	125	2.10	0.546	0.905	.366
Yes	225	2.05	0.586		
Using kerosene lamp					
No	321	2.06	0.568	−0.272	.786
Yes	29	2.09	0.619		
Using candles					
No	314	2.08	0.583	2.012	.050
Yes	36	1.92	0.448		
Wellbeing domain and source of power supply					
Using generators					
No	211	3.34	1.09	−0.017	.898
Yes	139	3.34	0.998		
Using battery power for lighting					
No	125	3.40	1.03	0.873	.383
Yes	225	3.30	1.06		
Using kerosene lamp					
No	321	3.34	1.04	−0.091	.928
Yes	29	3.35	1.20		
Using candles					
No	314	3.32	1.06	−0.812	.430
Yes	36	3.46	0.992		

An independent *t*-test was used to compare the means of shelter connection to mains electricity supply in terms of anxiety, depression and wellbeing. The results showed statistically significant differences in shelter connection to mains electricity supply and depression, and wellbeing domains (*p* = .000, .012, .020, respectively). Those who were living in shelters not connected to a mains electricity supply had elicited higher scores on anxiety and depression that those who had an electricity supply. Participants who had access to electricity also had higher wellbeing scores.

### Effect of reliability of electricity on mental health

An MANOVA test was used to compare the means of reliability of electricity supply regarding anxiety, depression and wellbeing (Table 3). The results showed statistically significant differences between the reliability of electricity supply and anxiety domains (*p* = .027), with a higher mean score (2.74) for households without an electricity supply than a household with a reliable or intermittent electricity supply (Table 3). Also, the results revealed statistically significant differences between the reliability of electricity supply and depression domain (*p* = .010), with a higher mean score (2.63) for households without an electricity supply than a household with a reliable or intermittent electricity supply (Table 3). However, the results showed that respondents who live in a household with a reliable electricity supply have a higher wellbeing score (3.49) than those with an intermittent supply or no electricity supply and there were statistically significant differences between the reliability of electricity supply and wellbeing domain (*p* = .050).

Although a difference between sources of the shelter power supply includes using generators, battery power for lighting, kerosene lamp and candles, the results showed no statistically significant differences between the source of electrical power and the anxiety, depression and wellbeing domains.

The differences in severity of Anxiety and depression regarding electricity bill, source of lighting, and reliability of bower Table (4) showed there was only a significant difference in the severity of anxiety and depression (c^2^ = 18.483, 8.341 and p-value = .014, .039 respectively) in regard to the amount of electricity bill, in which the severity of anxiety and depression decreased among participants paid bills less than 125 NIS.

**Table 4.** The relations between electricity bill, main source of lighting and reliability of power on anxiety and depression.

Categories	Severity of anxiety					Statistical analysis	
	Total	Mild (*n* = 24)	Moderate (*n* = 109)	Severe (*n* = 217)		χ^2^	*p*-value
Electricity bill							
⩽125	186 (53.1)	19 (79.1)	61 (56)	106 (48.8)		8.483	.014*
>125	164 (46.9)	5 (20.9)	48 (44)	111 (51.2)			
Main source of lighting							
Gas	5 (1.4)	0 (0)	1 (0.9)	4 (1.8)		7.659	.468
Electricity	317 (90.6)	21 (87.5)	100 (91.7)	196 (90.3)			
Generator	2 (0.6)	0 (0)	1 (0.9)	1 (0.5)			
Battery	13 (3.7)	0 (0)	4 (3.7)	9 (4.1)			
Solar panel	13 (3.7)	3 (12.5)	3 (2.8)	7 (3.2)			
Reliability of power: electricity supply reliable							
Reliable	57 (16.3)	4 (16.7)	20 (18.3)	33 (15.2)		1.221	.875
Intermittent	285 (81.4)	20 (83.3)	86 (78.9)	179 (82.5)			
No electricity supply	8 (2.3)	0 (0)	3 (2.8)	8 (2.3)			
	Severity of depression					Statistical analysis	
	Total	Mild (*n* = 24)	Moderate (*n* = 110)	Moderate to severe (*n* = 154)	Severe (*n* = 62)	χ2	*p*-value
Electricity bill							
⩽125	186 (53.1)	19 (79.2)	61 (56)	75 (48.7)	31 (50)	8.341	.039*
>125	164 (46.9)	5 (20.8)	48 (44)	79 (51.3)	31 (50)		
Main source of lighting							
Gas	5 (1.4)	0 (0)	1 (0.9)	4 (2.6)	0 (0)	13.885	. 308
Electricity	317 (90.6)	21 (87.5)	101 (91.8)	137 (89)	58 (93.5)		
Generator	2 (0.6)	0 (0)	1 (0.9)	1 (0.6)	0 (0)		
Battery	13 (3.7)	0 (0)	4 (3.6)	5 (3.2)	4 (6.5)		
Solar panel	13 (3.7)	3 (12.5)	3 (2.7)	7 (4.5)	0 (0)		
Reliability of power: electricity supply reliable							
Reliable	57 (16.3)	4 (16.7)	21 (19.1)	18 (11.7)	14 (22.6)	8.447	.207
Intermittent	285 (81.4)	20 (83.3)	86 (78.2)	134 (87)	45 (72.6)		
No electricity	8 (2.3)	0 (0)	3 (2.7)	2 (1.3)	3 (4.8)		

**Table:** The relations between electricity bill, main source of lighting and reliability of power on anxiety and depression.

	Electricity bill		Domain of anxiety	
	*r*	*p*-value	*r*	*p*-value
Domain of anxiety	.191	.000		
Domain of depression	.152	.004	.640	.000

### Effect of age and income on mental health

Finally, a Pearson correlation showed a significant positive relationship between age and anxiety (*R*(350) = 0.117, *p* < .05), and as expected Age and Income (*R*(349) = 0.402, *p* < .001).

Income was also significantly correlated with both anxiety (R(349) = −0.20, *p* < .001), and depression (*R*(349) = −0.16, *p* < 0.001), with both decreasing as income levels increased.

Different aspects of everyday activities are affected by the lack of electricity and might contribute to stress such as in activities involving the education of children, cooking and cleaning. The majority of respondents have children below 18 years old, and also 80% have children who go to school. Respondents indicated that students have difficulties in doing homework due to the lack and shortage of electricity.

## Discussion

The population of Gaza suffers from a prolonged and constant deficit of electricity. The research has revealed the significant impacts of electricity access on the mental health of the residents. Furthermore, the current study demonstrates the impact on wellbeing of a lack of basic amenities such as heating and access to a computer. Additionally, the recent studies showed a significant association between income and mental health, which supports the plethora of studies that make associative and causal links between poverty and poor mental health (e.g., Lund et al., 2010, Ridley et al., 2020).

Energy access and associated poverty is a determinant of health in low- and middle-income countries (LMICs) and can impact on social and community networks in many ways, including access to suitable environments to study, functional health care services, communication and employment. Importantly, access to reliable, non-polluting and affordable energy can support employment, promote economic development and help address poverty (Bruce & Ding, 2014). The novelty of our research is the focus on mental health needs in conflict and post-conflict LMIC settings by emphasising the role that daily stressors play in mediating direct war exposure and mental health outcomes. The continual, systemic and structural oppression, which is psychologically no less detrimental than conflict and violence (Hammad & Tribe, 2020), is aggravated by the lack of electricity. ‘Bundled hardships’ of energy poverty in association with financial strain, heat stress and other factors can exacerbate mental health effects. The physical and mental health impacts of living in energy poverty may be acute or chronic and addressing this is a matter of social and environmental justice, particularly as the effects of climate change become increasingly apparent and issues such as heat stress disproportionately affect poorer people in LMICs (Jessel et al., 2019).

There is a number of limitations to our study. First, we are aware that electricity use may well be a proxy measure for other conditions relating to housing/location/finances and so forth, and if this is the case this could partly confound the results. Furthermore, access to addresses of certain households was not always any easy (or even a possible) task, and hence location and accessibility may well have in some way biased the sample that was used.

Access to basic services is a human rights issue, and one with profound consequences. Our findings add to recent research in Gaza which found a sense of hopelessness and distress among students. In response to living under siege and with the associated poor living conditions, the authors found that a loss of resilience and hope impacted mental distress, including anxiety, traumatic stress and depression with male students found to have a particularly high suicide risk (Veronese et al., 2021). Thus, the research findings linking access to energy with mental health and psychological and social resilience help to understand how improvement of access to electricity can increase people’s capacities to maintain mental wellbeing.

Further research is needed on how the lack of access to electricity affects children’s mental health and their educational outcomes. Studies demonstrate that children and adolescents living in Palestine are exposed to high levels of traumatic experiences that impact the prevalence of mental health issues (Dimitry, 2012). It would be beneficial to demonstrate how making electricity more reliable and accessible can help mitigate these issues.

It is crucial to investigate how the issues in accessing electricity can further affect people receiving timely and effective healthcare in Gaza and other conflict-affected areas considering the colossal damage to the system due to the recent cycle of Israeli military aggression (Asi et al., 2021). The incidents of the death, injury, and ill health of people from lack of access to electricity in Gaza are usually beyond the international spotlight. This research highlights the urgent importance of incorporating access to affordable, consistent and sustainable energy and increasing opportunities for well-paid employment in humanitarian responses. There is also an urgent need for a systematic approach to collecting and reporting data on access to electricity and electric appliances in such settings, along with recognition of access to electricity as a public health issue.

## Supplemental Material

sj-docx-1-isp-10.1177_00207640231194479 – Supplemental material for The impact of access to electricity on mental health in conflict-affected territories: An exploratory study in Gaza Click here for additional data file. Supplemental material, sj-docx-1-isp-10.1177_00207640231194479 for The impact of access to electricity on mental health in conflict-affected territories: An exploratory study in Gaza by Mazen AbuQamar, Dalia Eltayyan, Irina Kuznetsova, Surindar Dhesi, Jonathan Catling, Raya AL-Dadah, Mahmoud Saad and Mohammad Abuhaiba in International Journal of Social Psychiatry

## References

[bibr1-00207640231194479] Abu-Rmeileh N. M. Hammoudeh W. Mataria A. Husseini A. Khawaja M. Shannon H. S. Hogan D. Watt G. C. Zurayk H. Giacaman R. (2012). Health-related quality of life of Gaza Palestinians in the aftermath of the winter 2008–09 Israeli attack on the Strip. The European Journal of Public Health, 22(5), 732–737. 23012310 10.1093/eurpub/ckr131 PMC3857916

[bibr2-00207640231194479] Asi Y. M. Tanous O. Wispelwey B. AlKhaldi M. (2021). Are there ‘two sides’ to attacks on healthcare? Evidence from Palestine. European Journal of Public Health, 31(5), 927–928. 10.1093/eurpub/ckab167 34562003 PMC8546872

[bibr3-00207640231194479] Beard C. Hsu K. J. Rifkin L. S. Busch A. B. Björgvinsson T. (2016). Validation of the PHQ-9 in a psychiatric sample. Journal of Affective Disorders, 193, 267–273. 26774513 10.1016/j.jad.2015.12.075

[bibr4-00207640231194479] Braubach M. (2011). Key challenges of housing and health from WHO perspective. International Journal of Public Health, 56(6), 579–580. 10.1007/s00038-011-0296-y 21894569

[bibr5-00207640231194479] Bruce N. Ding C. (2014). Health benefits from energy access in LMICs: Mechanisms, impacts, and policy opportunities. In Halff A. Ovacool B. K. Rozhon J. (Eds.), Energy poverty: Global challenges and local solutions (pp. 106–132). Oxford University Press. https://oxford.universitypressscholarship.com/view/10.1093/acprof:oso/9780199682362.001.0001/acprof-9780199682362-chapter-6

[bibr6-00207640231194479] Dimitry L. (2012). A systematic review on the mental health of children and adolescents in areas of armed conflict in the Middle East. Child: Care, Health and Development, 38(2), 153–161. 10.1111/j.1365-2214.2011.01246.x 21615769

[bibr7-00207640231194479] Hamad B. A. Jones N. Samuels F. (2020). Mental health and psychosocial challenges facing adolescent girls in conflict-affected settings: The case of the Gaza Strip. Arab Journal of Psychiatry, 31(2), 169–180.

[bibr8-00207640231194479] Hammad J. Tribe R. (2020). Social suffering and the psychological impact of structural violence and economic oppression in an ongoing conflict setting: The Gaza Strip. Journal of Community Psychology, 48(6), 1791–1810. 32399970 10.1002/jcop.22367

[bibr9-00207640231194479] Ibrahim A. Hor S. Bahar O. S. Dwomoh D. McKay M. M. Esena R. K. Agyepong I. A. (2016). Pathways to psychiatric care for mental disorders: A retrospective study of patients seeking mental health services at a public psychiatric facility in Ghana. International Journal of Mental Health Systems, 10, 1–11. 27729938 10.1186/s13033-016-0095-1 PMC5048657

[bibr10-00207640231194479] International Renewable Energy Agency. (2021). Tracking SDG 7: The energy progress report. https://www.irena.org/publications/2021/Jun/Tracking-SDG-7-2021

[bibr11-00207640231194479] Irwin B. R. Hoxha K. Grépin K. A. (2020). Conceptualising the effect of access to electricity on health in low-and middle-income countries: A systematic review. Global Public Health, 15(3), 452–473. 31770072 10.1080/17441692.2019.1695873

[bibr12-00207640231194479] Ismail M. Moghavvemi M. Mahlia T. (2013). Energy trends in Palestinian territories of West Bank and Gaza Strip: Possibilities for reducing the reliance on external energy sources. Renewable and Sustainable Energy Reviews, 28, 117–129. 10.1016/j.rser.2013.07.047

[bibr13-00207640231194479] Jessel S. Sawyer S. Hernández D. (2019). Energy, poverty, and health in climate change: A comprehensive review of an emerging literature. Frontiers in Public Health, 7, 357. 10.3389/fpubh.2019.00357 31921733 PMC6920209

[bibr14-00207640231194479] Kocalevent R. D. Hinz A. Brähler E. (2013). Standardization of the depression screener patient health questionnaire (PHQ-9) in the general population. General Hospital Psychiatry, 35(5), 551–555. 23664569 10.1016/j.genhosppsych.2013.04.006

[bibr15-00207640231194479] Kroenke K. Strine T. W. Spitzer R. L. Williams J. B. W. Berry J. T. Mokdad A. H. (2009). The PHQ-8 as a measure of current depression in the general population. Journal of Affective Disorders, 114(1–3), 163–173. 10.1016/j.jad.2008.06.026 18752852

[bibr16-00207640231194479] Löwe B. Decker O. Müller S. Brähler E. Schellberg D. Herzog W. Herzberg P. Y. (2008). Validation and standardization of the Generalized Anxiety Disorder Screener (GAD-7) in the general population. Medical Care, 46, 266–274. 18388841 10.1097/MLR.0b013e318160d093

[bibr17-00207640231194479] Lund C. Breen A. Flisher A. J. Kakuma R. Corrigall J. Joska J. A. Swartz L. Patel V. (2010). Poverty and common mental disorders in low and middle income countries: A systematic review. Social Science & Medicine, 71(3), 517–528. 20621748 10.1016/j.socscimed.2010.04.027 PMC4991761

[bibr18-00207640231194479] Madianos M. G. Sarhan A. L. Koukia E. (2011). Major depression across West Bank: A cross-sectional general population study. International Journal of Social Psychiatry, 58(3), 315–322. 10.1177/0020764010396410 21441281

[bibr19-00207640231194479] Makkawi I. (2015). Community psychology enactments in Palestine: Roots and current manifestations. Journal of Community Psychology, 43(1), 63–75.

[bibr20-00207640231194479] Manea L. Gilbody S. McMillan D. (2012). Optimal cut-off score for diagnosing depression with the Patient Health Questionnaire (PHQ-9): A meta-analysis. CMAJ, 184(3), E191–E196. 10.1503/cmaj.110829 PMC3281183 22184363

[bibr21-00207640231194479] McNeely C. Barber B. K. Spellings C. Giacaman R. Arafat C. Daher M. El Sarraj E Abu Mallouh M. (2014). Human insecurity, chronic economic constraints and health in the occupied Palestinian territory. Global Public Health, 9(5), 495–515. 24766078 10.1080/17441692.2014.903427

[bibr22-00207640231194479] OCHA. (n.d.). Electricity in the Gaza Strip. https://www.ochaopt.org/page/gaza-strip-electricity-supply

[bibr23-00207640231194479] Ridley M. Rao G. Schilbach F. Patel V. (2020). Poverty, depression, and anxiety: Causal evidence and mechanisms. Science, 370(6522), eaay0214. 10.1126/science.aay0214 33303583

[bibr24-00207640231194479] Singh A. Daniel L. Baker E. Bentley R. (2019). Housing disadvantage and poor mental health: A systematic review. American Journal of Preventive Medicine, 57(2), 262–272. 31326010 10.1016/j.amepre.2019.03.018

[bibr25-00207640231194479] Spitzer R. L. Kroenke K. Williams J. B. W. Löwe B. (2006). A brief measure for assessing generalized anxiety disorder the GAD-7. Archives of Internal Medicine, 166, 1092–1097. 16717171 10.1001/archinte.166.10.1092

[bibr26-00207640231194479] Thabet A. A. Thabet S. (2015). Stress, trauma, psychological problems, quality of life, and resilience of palestinian families in the Gaza strip. Clinical Psychiatry, 1(2), 1–6. https://www.primescholars.com/articles/stress-trauma-psychological-problemsquality-of-life-and-resilience-of-palestinianfamilies-in-the-gaza-strip-104682.html

[bibr27-00207640231194479] Topp C. W. Østergaard S. D. Søndergaard S. Bech P. (2015). The WHO-5 Well-Being Index: A systematic review of the literature. Psychotherapy and Psychosomatics, 84(3), 167–176. 25831962 10.1159/000376585

[bibr28-00207640231194479] Veronese G. Pepe A. Diab M. Abu Jamey Y. Kagee A. (2021). Living under siege: Resilience, hopelessness, and psychological distress among Palestinian students in the Gaza Strip. Global Mental Health, 8, e40. 10.1017/gmh.2021.37 PMC8518025 34703614

[bibr29-00207640231194479] World Bank Open Data. (n.d.). Population, total: West Bank and Gaza. https://data.worldbank.org/indicator/SP.POP.TOTL?locations=PS

[bibr30-00207640231194479] Ziersch A. Due C. (2018). A mixed methods systematic review of studies examining the relationship between housing and health for people from refugee and asylum seeking backgrounds. Social Science & Medicine, 213, 199–219. 10.1016/j.socscimed.2018.07.045 30142501

