# Supplementary material for: The impact of access to electricity on mental health in conflict-affected territories: An exploratory study in Gaza
Source: Int J Soc Psychiatry. 2023 Sep 7;69(8):2148–56. doi: 10.1177/00207640231194479 (PMC10685693; doi:10.1177/00207640231194479)
Supplement: sj-docx-1-isp-10.1177_00207640231194479 – Supplemental material for The impact of access to electricity on mental health in conflict-affected territories: An exploratory study in Gaza [file sj-docx-1-isp-10.1177_00207640231194479.docx]

**Supplementary materials**

**Table 1: Demographic characteristics of respondents**

| **Variables: *n*=350** | Frequency | Percentage % |
| --- | --- | --- |
| **Gender** | | |
| Male | 172 | 49 |
| Female | 198 | 51 |
| **Governorate** |  |  |
| NG | 71 | 20.3 |
| G | 134 | 38.3 |
| MG | 26 | 7.4 |
| KH | 73 | 20.9 |
| RG | 46 | 13.1 |
| **Age** | | |
| 18-24 years | 54 | 15.4 |
| 25-29 years | 40 | 11.4 |
| 30–34 years | 64 | 18.3 |
| 35–39 years | 61 | 17.4 |
| 40–44 years | 47 | 13.4 |
| 45-49 years | 36 | 10.3 |
| 50-59 years | 33 | 9.4 |
| 60 and older | 15 | 4.3 |
| **Marital status** | | |
| Never married | 74 | 21.1 |
| Married or living together | 223 | 63.7 |
| Divorced/separated | 29 | 8.3 |
| Widowed | 24 | 6.9 |
| **Qualification** |  |  |
| Illiterate | 18 | 5.1 |
| primary school (elementary) | 6 | 1.7 |
| Middle school (preparatory) | 11 | 3.1 |
| High school certificate (secondary) | 55 | 15.7 |
| Intermediate Post high school diploma | 57 | 16.3 |
| Graduation (BA) | 140 | 40 |
| Professional degree, MA and above | 63 | 18 |
| **Employment** |  |  |
| Employed full time | 145 | 41.4 |
| Employed part-time | 45 | 12.9 |
| Retired | 6 | 1.7 |
| Not working | 6 | 1.7 |
| Student | 55 | 15.7 |
| Unemployed | 48 | 13.7 |
| Maternity leave | 2 | .6 |
| Housewife | 43 | 12.3 |
| **Income** |  |  |
| Absolute Poverty | 239 | 68.3 |
| Relative Poverty | 34 | 9.7 |
| non-poverty | 77 | 22.0 |
| **Existence of chronic disease** | | |
| Yes | 114 | 32.6 |
| No | 236 | 67.4 |
